# Cardiac and Pulmonary Rehabilitation: Two Underutilized Approaches with Some Unexpected Benefits

**DOI:** 10.3390/jcm12082847

**Published:** 2023-04-13

**Authors:** Pasquale Ambrosino, Giuseppina Marcuccio, Roberto Formisano, Laura Marcuccio, Rosanna Filosa, Mauro Maniscalco

**Affiliations:** 1 Directorate of Telese Terme Institute, Istituti Clinici Scientifici Maugeri IRCCS, 82037 Telese Terme, Italy; 2 Pulmonary Rehabilitation Unit of Telese Terme Institute, Istituti Clinici Scientifici Maugeri IRCCS, 82037 Telese Terme, Italy; giuseppina_marcuccio@hotmail.it (G.M.); rfilosa@unisannio.it (R.F.); 3 Cardiac Rehabilitation Unit of Telese Terme Institute, Istituti Clinici Scientifici Maugeri IRCCS, 82037 Telese Terme, Italy; roberto.formisano@icsmaugeri.it; 4 Neurological Rehabilitation Unit of Telese Terme Institute, Istituti Clinici Scientifici Maugeri IRCCS, 82037 Telese Terme, Italy; laura.marcuccio@icsmaugeri.it; 5 Department of Science and Technology, University of Sannio, 82100 Benevento, Italy; 6 Department of Clinical Medicine and Surgery, Federico II University, 80131 Naples, Italy

Although still underutilized [1,2], exercise-based rehabilitation has become an integral component in the management of chronic cardiovascular and respiratory diseases, thus being incorporated into national and international guidelines for various clinical conditions [3]. In this regard, a large amount of scientific data supports the beneficial effects of this multidisciplinary approach in terms of morbidity and mortality, with a significant improvement in autonomy and the quality of life, reduced disability and greater participation in social activities [4]. However, both cardiac and pulmonary rehabilitation programs are generally planned and designed only for a limited number of index diagnoses [5]. Thus, if chronic obstructive pulmonary disease (COPD) is the index disease for pulmonary rehabilitation [6], heart failure with reduced ejection fraction and coronary artery disease are the target clinical conditions for a Class I recommendation for cardiac rehabilitation [7,8]. Consequently, a number of other orphan diseases (e.g., idiopathic pulmonary fibrosis, bronchiectasis, heart failure with preserved ejection fraction), are not univocally considered in clinical guidelines for multidisciplinary rehabilitation, despite having a high epidemiological impact and serious burden on healthcare systems [5].

In this scenario, the new severe acute respiratory syndrome coronavirus 2 (SARS-CoV-2) has generated a further potential element of disability in a growing and increasingly long-lived world population [9], thus increasing the demand for new therapeutic approaches able to manage a new level of clinical complexity. Multidisciplinary rehabilitation has immediately emerged as one the most suitable strategies to satisfy this increased need for assistance after the acute phase of coronavirus disease 2019 (COVID-19) [10]. Indeed, evidence of its beneficial effects in this new clinical setting has rapidly multiplied, and rehabilitation has proven to be a safe and feasible approach after hospital discharge due to its ability to improve a number of relevant clinical outcomes related to lung function, exercise capacity, quality of life and mental health [11]. Of course, specifically designed programs for COVID-19 patients were not available and, pending guidelines dedicated to this new clinical condition, most of the authors have borrowed or adapted pulmonary rehabilitation programs designed for other pathologies [12]. An interesting lexical analysis of the literature showed that rehabilitation in patients with COVID-19 has been focused more on exercises and physical training for the recovery of multiorgan disability and motor impairments, rather than on lung disease [13]. Thus, while providing a comprehensive literature overview, the authors highlighted a discrepancy between the rehabilitation programs proposed for COVID-19 patients and those usually delivered to patients with interstitial lung disease of different etiology.

Interestingly, pulmonary rehabilitation has also been proven to deliver some unexpected benefits to convalescent COVID-19 patients. Literature evidence suggests that endothelial dysfunction may play a key role in the pathogenesis of COVID-19 and its residual manifestations [14]. In fact, SARS-CoV-2 has the potential to directly infect vascular endothelial cells [15], while also inducing an inflammatory-mediated loss of their functional integrity [16]. Accordingly, it has been shown that impaired endothelial function in convalescent COVID-19 patients may be somehow associated with a reduced ventilatory efficiency at cardiopulmonary exercise tests [17]. Moreover, based on meta-analytical data, a direct association has been documented between reduced endothelial function and the presence of post-acute sequalae of COVID-19 [18]. In this scenario, pulmonary rehabilitation has been shown to significantly and consistently restore endothelium-dependent flow-mediated dilatation (FMD) in COVID-19, with even a link between endothelial function and lung function improvements [19]. Therefore, considering that endothelial dysfunction is the earliest stage of atherosclerosis and that FMD is a surrogate marker of cardiovascular risk [20,21], it has been hypothesized that exercise-based rehabilitation may help reduce the increased cardiovascular burden of COVID-19 reported in large epidemiological studies [22].

This is by no means novel considering that a growing number of scientific articles seem to be moving in this direction, showing a beneficial effect of cardiac and pulmonary rehabilitation on vascular health and, potentially, on the increased cardiovascular risk [23]. In randomized controlled trials of COPD patients, despite the occasionally conflicting evidence [24,25], pulmonary rehabilitation proved to restore FMD and reactive hyperemia index, as measured at peripheral artery tonometry, especially in more severe disease [26,27,28]. An interesting article by Lanza et al. [29] summarized the literature evidence on the effects of cardiac rehabilitation on endothelial function in different clinical settings, including heart failure, stable coronary artery disease, and acute myocardial infarction. The authors concluded that, based on the available data, supervised exercise delivered as part of a rehabilitation program might be able to achieve an adequate volume and intensity to provide this beneficial effect in terms of improved endothelial function and reduced cardiovascular risk.

Collectively, these data highlight the pleiotropic and unexpected benefits that rehabilitation can bring to the management of a number of clinical conditions, thus focusing attention on the need for more high-quality evidence on the usefulness of rehabilitation in diseases for which the strength of recommendation is still weak. Among these, interstitial lung disease is one of the airway diseases with low/moderate and (sometimes) conflicting evidence and, therefore, a weak recommendation for this multidisciplinary approach [30,31]. However, based on recent meta-analytical data, long-lasting improvements in functional exercise capacity, dyspnea and quality of life are to be expected in this clinical setting after rehabilitation [32]. A recent study of 28 patients with fibrotic interstitial lung disease analysed the clinical features that are able to predict the effectiveness of pulmonary rehabilitation, concluding that it should be offered soon after the disease is diagnosed and before the functional impairment becomes severe [33]. Accordingly, another study on a similar patient population investigated the benefits of add-on pulmonary rehabilitation in patients treated with antifibrotic drugs [34]. Interestingly, at the 3-month follow-up, the authors documented significantly less dyspnea, with a better quality of life and exercise capacity, as measured by the 6 min walking test (6MWT), in participants who received the combined approach compared to those treated with antifibrotic drugs alone. Based on meta-analytical data, similar results on similar outcomes are to be expected after pulmonary rehabilitation in other non-COPD diseases, including bronchiectasis [35] and pulmonary hypertension [36].

Like pulmonary rehabilitation, cardiac rehabilitation is currently limited to a relatively low number of clinical conditions in most guidelines, including coronary artery disease, heart failure with reduced ejection fraction, and peripheral artery disease [5]. Literature evidence unequivocally supports the ability of this multidisciplinary approach to reduce mortality after myocardial infarction by up to 25%, while reducing the risk of hospital readmission by 18% [37]. Further improved results regarding mortality, hospitalization, incident stroke, and atrial fibrillation have been reported when cardiac rehabilitation is delivered to patients with heart failure [38,39]. Moreover, while confirming its capacity to reduce overall disability, cardiac rehabilitation has been shown to result in a significant improvement in exercise tolerance and adaptations to exercise stress, as expressed by its impact on metabolic equivalent of the task, maximal oxygen consumption, and 6MWT score [40]. Accordingly, when monitoring echocardiographic parameters in patients with acute myocardial infarction, cardiac rehabilitation has been able to positively impact the left ventricular diastolic function, which is known to be associated with exercise tolerance [41]. Peripheral artery disease is another prototypical clinical condition for which exercise-based interventions are critical to achieve good disease control [42]. Therefore, supervised exercise is highly recommended in this clinical setting (Class I, Level of Evidence A), with the possibility of proposing a home-based exercise program as an alternative (Class IIa, Level of Evidence A) [43]. An interesting article by Anghel et al. [44] summarized the literature evidence on the plethora of exercise-based approaches available for these patients, concluding that exercise in mild-to-moderate claudication can be standalone or delivered as part of a structured cardiac rehabilitation program, with a minimum 12 weeks duration to improve functional capacity and the quality of life.

Alongside these clinical conditions for which the evidence is solid and numerous, there are others for which the data on cardiac rehabilitation are scarce and not univocal. The 2022 American College of Cardiology/American Heart Association guidelines highlight this paucity for heart failure with preserved or mildly reduced ejection fraction, prioritizing the need for high-quality randomized controlled trials on cardiac rehabilitation over small studies mainly focused on exercise training [45]. Similarly, cardiac rehabilitation is recommended by the European Society of Cardiology Working Groups only for selected patients after surgical aortic valve replacement, but not for the management of patients after transcatheter aortic valve implantation (TAVI) [46]. However, the rehabilitation approach has shown efficacy in improving exercise capacity, muscle strength, and the quality of life of TAVI patients, although these benefits appear to diminish after 1 year [47]. These short-term functional benefits are also confirmed by meta-analytical data [48], thus questioning the need for further randomized controlled studies with a longer follow-up to fully evaluate the cost-effectiveness of cardiac rehabilitation in this patient population.

Taken together, these data appear to support the enormous, and often unexpected, benefits that rehabilitation and exercise-based interventions can offer to a long list of clinical conditions, some of which are not exactly considered a preferred target for this approach. The mechanisms underlying these potential benefits, in terms of functional capacity, quality of life, rehospitalizations, and mortality, are multiple and still poorly understood, with a potential major role in the control of inflammation and endothelial dysfunction. Although rehabilitation programs are usually designed for single pathologies, it should be considered that a patient is rarely affected by isolated clinical conditions, as they are often interconnected and tend to coexist in the same individual. Therefore, the new paradigm of rehabilitation directed against multimorbidity should be introduced and considered when developing clinical recommendations and designing randomized controlled trials on the utility of this exercise-based approach in different clinical settings. In this scenario, it should be considered that the recent SARS-CoV-2 pandemic has given a significant boost to the digitization process and to tele-assisted medicine in several Countries, and it is reasonable to assume that telerehabilitation could contribute to satisfying the growing unmet needs of rehabilitation services in the future.
